# Self-Test Procedures for Gas Sensors Embedded in Microreactor Systems

**DOI:** 10.3390/s18020453

**Published:** 2018-02-03

**Authors:** Andreas Helwig, Angelika Hackner, Gerhard Müller, Dario Zappa, Giorgio Sberveglieri

**Affiliations:** 1 Airbus Central R&T, D-81663 Munich, Germany; andreas.helwig@airbus.com (A.He.); angelika.hackner@airbus.com (A.Ha.); 2 Department of Applied Sciences and Mechatronics, Munich University of Applied Sciences, D-80335 Munich, Germany; 3 SENSOR, Dipartimento di Ingegneria dell’Informazione, Università degli Studi di Brescia, via Valotti 9, 25123 Brescia, Italy; dario.zappa@unibs.it (D.Z.); giorgio.sberveglieri@unibs.it (G.S.)

**Keywords:** metal oxide, gas sensor, drift, poisoning, self-test, MEMS, micro-reactor, micro-heater

## Abstract

Metal oxide (MOX) gas sensors sensitively respond to a wide variety of combustible, explosive and poisonous gases. However, due to the lack of a built-in self-test capability, MOX gas sensors have not yet been able to penetrate safety-critical applications. In the present work we report on gas sensing experiments performed on MOX gas sensors embedded in ceramic micro-reaction chambers. With the help of an external micro-pump, such systems can be operated in a periodic manner alternating between flow and no-flow conditions, thus allowing repetitive measurements of the sensor resistances under clean air, $R_{0}$, and under gas exposure, $R_{gas}$, to be obtained, even under field conditions. With these pairs of resistance values, eventual drifts in the sensor baseline resistance can be detected and drift-corrected values of the relative resistance response $Resp=\left(R_{0}-R_{gas}\right)/R_{0}$ can be determined. Residual poisoning-induced changes in the relative resistance response can be detected by reference to humidity measurements taken with room-temperature-operated capacitive humidity sensors which are insensitive to the poisoning processes operative on heated MOX gas sensors.

## 1. Introduction

Resistive metal oxide (MOX) gas sensors are low-cost sensors which are sensitive to a wide variety of toxic and combustible gases [1,2,3,4,5,6]. As such they are potentially useful in a wide range of fields, including climate-, safety-, security-, and process monitoring. A second attractive feature is that intensive miniaturization approaches have driven the electrical power consumption of such sensors to attractively low levels. To date, advances in micro-electro-mechanical-systems (MEMS) micro-heater technologies [7,8,9,10,11], methods of non-stationary sensor operation [12,13,14,15], and clever use of self-heating effects in MOX nanowire assemblies [16,17,18,19,20] have lowered the power consumption of MOX gas sensors to levels in the range of several tens of micro-watts. Such levels open up perspectives towards self-powering sensor units, comprising energy harvesting and wireless interrogation functionalities alongside the MOX gas sensors themselves.

Problems that have been well-known throughout the entire development of the MOX gas sensor field are that MOX sensors suffer to varying degrees from the effects of baseline resistance drift and from poisoning interactions which make heated MOX surfaces less and less accessible to reactive gases. As during sensor operation such processes are usually slow, and as these do not principally compromise the capability of MOX sensors to detect gases as such, the underlying mechanisms of sensor degradation have not been the subject of intense scientific investigation [21,22,23,24,25,26,27,28,29,30]. Such degradation processes, however, have always remained a problem of practical concern and precluded MOX gas sensors and sensor arrays from penetrating fields where quantitative measurements need to be made or where safety-critical alarm functionalities are to be provided [31,32]. Attempting to ameliorate this situation, we show in this paper how the effects of sensor degradation can be detected and partially corrected to attain higher levels of signal quality, also under conditions of practical field operation.

In the first part of the paper we consider poisoning interactions of Hexamethyldisiloxane (HMDS) on heated MOX surfaces. In this context, HMDS is taken as a reference substance standing for all kinds of poisons that tend to form passivating overlayers on heated MOX surfaces (silanes, silicones, silicates, lead oxide film formers, sulphides, phosphates, and organic halides [21,22,25,26,27,28,30]. These first considerations show that HMDS poisoning can have a large impact both on the magnitude of the sensor baseline resistance $R_{0}$ in clean air and on the gas-induced resistance changes $∆R=R_{0}-R_{gas}$. Secondly, as $R_{0}$ and ∆R scale in a similar way, it is shown that the value of the relative resistance response $Resp=∆R/R_{0}$ is a much better indicator of the momentary gas concentrations $c_{gas}(t)$. Our poisoning experiments, thirdly, show that the normalization inherent in the calculation of Resp is not sufficient to remove all effects of sensor degradation. In particular, it is shown that HMDS poisoning has a larger effect on Resp when the analyte molecules have large as opposed to small molecular masses. Whereas all these effects are easy to assess under laboratory conditions where control signals of a gas test rig are available to assess the resistances $R_{0}$ before and $R_{gas}$ after test gas exposures, this is not normally possible under field conditions where the sensors are continually exposed to the environment. 

In the second part of the paper, we show that such control signals for the evaluation of Resp can also be provided under field conditions when MOX gas sensors are embedded into tiny micro-reaction chambers. Such micro-reactor systems allow the embedded gas sensors to be periodically operated under reference air and test gas conditions, thus providing pairs of measurements of $R_{0}$ and $R_{gas}$ at closely similar times $t_{i}$ and $t_{i+1}$, with ∆t being small enough to exclude any sizeable drifts in $R_{0}$ and ∆R during that time. With these pairs of $R_{0}$ and ∆R values, drift-corrected values of Resp can be obtained. 

In the final part of the paper we show that the residual effects of HMDS poisoning on Resp, which do not drop out in the drift correction process, can be detected by reference measurements with capacitive humidity sensors. These latter measurements make use of the changing humidity levels in practically all application environments which makes water vapor an ideal and freely available reference gas. The key value of capacitive humidity sensors is that these are room-temperature-operated and thus unable to provide the thermal energy input needed to transform heavily sticking adsorbates into dense, solid passivation layers. Capacitive humidity sensors therefore are ideally suited to detect poisoning interactions on heated MOX surfaces.

A brief discussion on the current state of the art, possible future developments and optimization issues completes this paper.

## 2. Sensor Poisoning

In order to demonstrate the effects of sensor poisoning, we exposed SnO_2_ gas sensors to HMDS passivation treatments, which is a specific form of sensor poisoning and which contributes to sensor drift. These sensor materials, which were produced at the University of Brescia, were deposited onto ceramic heater substrates with pre-deposited Pt backside heater meanders by the high-temperature vapor transportation and condensation technique [33,34,35]. More details about the sensor preparation can be found in Appendix A. In order to assess the effects of passivation, the sensors were exposed to sequences of test gas pulses both in the state before passivation and after different-length HMDS treatments. The passivation itself was performed by guiding streams of synthetic air (SA) at a rate of 50 sccm/min through a bubbler filled with liquid HMDS [28]. The resulting HMDS vapors were then guided over the sensors for fixed amounts of time while being heated to their normal sensor operation temperature (T≈350 °C). Both sensor passivation and sensor performance assessments were performed at AIRBUS Innovation Works using a custom-designed gas test rig. 

**Figure 1:**
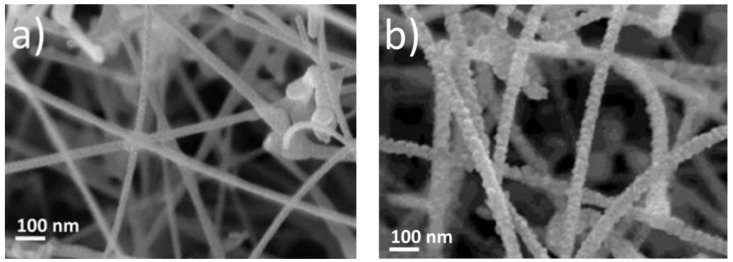
Scanning electron microscopy (SEM) pictures of SnO_2_ nanowire sensors (**a**) before and (**b**) after 1000 s of Hexamethyldisiloxane (HMDS) passivation.

**Figure 2:**
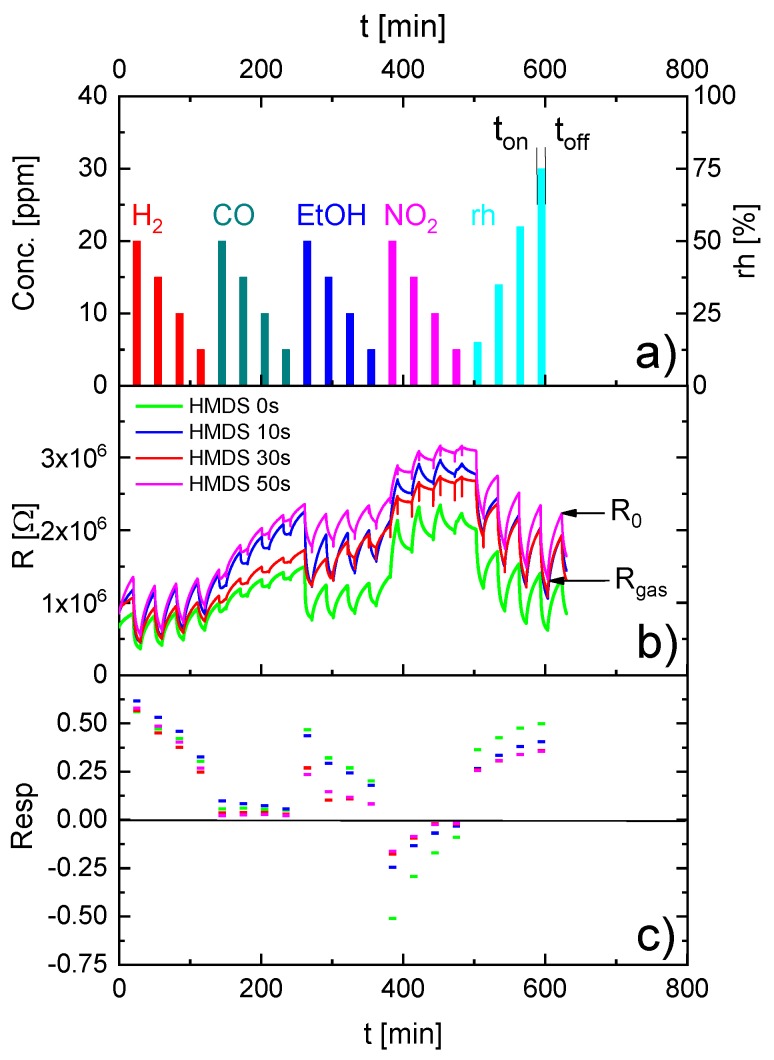
(**a**) Test gas exposure sequence. Test gases were supplied in gas flows of 200 sccm/min of dry synthetic air (SA); (**b**) gas-induced resistance changes; (**c**) relative resistance responses after different-length HMDS treatments.

The results obtained in this way are shown in Figure 1 and Figure 2. The first figure shows scanning electron microscopy (SEM) images of the SnO_2_ nanowire sensors both in their as-prepared states (Figure 1a) and after HMDS passivation (Figure 1b). A comparison of both images shows that HMDS passivation forms small particles of SiO_2_ on top of the SnO_2_ nanowires. Figure 1b, in particular, shows that the ensuing HMDS deposits form overlayers with a granular morphology, which more and more restrict the access of reactive gases to the SnO_2_ surfaces as HMDS treatment times are prolonged. Similar HMDS treatments performed on different kinds of commercial thick-film gas sensors consistently produced sensor resistance reductions as HMDS treatment times were prolonged. These reductions likely arise from the consumption of surface oxygen ions as HMDS adsorbates are converted into solid SiO_2_ overlayers. As we consistently see resistance enhancements when HMDS treatments are applied to nanowire assemblies, a second and more dominant form of deterioration needs to be assumed. Likely this second deterioration process results from a reduction in the number of wire-to-wire contacts and concomitantly in a smaller number of conducting percolation paths through the nanowire assemblies.

Figure 2 shows the results of gas measurements on one of these sensors after different degrees of HMDS passivation. The test gases for the assessment of the sensor performance were H_2_, CO, ethanol (EtOH), NO_2_ and water vapor at the concentrations given in Figure 2a. The ensuing changes in sensor resistance are presented in Figure 2b. One can observe that irrespective of the length of the HMDS treatments, the sensor continues to respond to all test gases. A brief look at the entirety of the data, however, clearly demonstrates that a certain value of sensor resistance R cannot be uniquely associated with the corresponding gas concentration. A closer look at this same data set reveals two important trends: (i) in data sets acquired at a constant level of HMDS passivation the sensor consistently drifts due to the fact it is not able to fully recover to baseline before any new gas exposure pulse is applied; (ii) comparing time series of sensor resistances acquired at different levels of HMDS passivation the same kind of resistance variation over time can be observed, however, with the average resistance level increasing after each passivation step. Figure 2c shows the relative resistance responses $Resp=\left[R_{0}\left(t_{on}\right)-R_{gas}\left(t_{off}\right)\right]/R_{0}\left(t_{on}\right)$ derived from the data of Figure 2b. Comparing the Resp data to the test gas sequences in Figure 2a, it can be seen that the Resp values scale in a much more consistent manner with the individual gas concentrations than the directly observed resistance values. Resp calculations, obviously, tend to remove the effects of concentration pile-ups, either on the sensor surface or in the sensor periphery, which occur when successive gas exposure pulses are applied before the sensor has been able to fully recover to baseline again. As a second effect the data in Figure 2c also vividly demonstrate that the process of Resp calculation does not remove the effects of sensor passivation. After some molecule-specific start-up effects, the Resp values of all gases do decrease as HMDS treatment times are prolonged. This is particularly the case when relatively massive analytes such as CO, EtOH or NO_2_ are considered. H_2_, in contrast is hardly affected by the effects of passivation. H_2_O with its moderate molecular mass is in between the extremes of H_2_ and EtOH or NO_2_, respectively. As the H_2_O response is sensitive to the passivation and as H_2_O vapor is present in large and largely variable concentrations in practically every application environment, H_2_O is a potentially very useful and freely available reference gas. 

**Figure 3:**
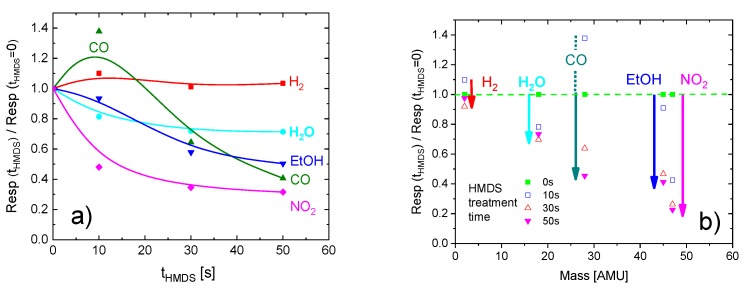
(**a**) Change in relative resistance response Resp with HMDS treatment time for analytes with different molecular masses; (**b**) change in relative resistance response with analyte molecular mass after different-length HMDS treatments. Analyte concentrations were 20 ppm (H_2_, EtOH, NO_2_) and rh = 80%, respectively. Arrows indicate losses in relative resistance response with HMDS passivation.

For the convenience of readers, the impacts of sensor passivation on the magnitude of the Resp values are quantitatively displayed in Figure 3a,b.

Summarizing, this first set of measurements shows that signatures of sensor drift and sensor poisoning are changes in the baseline resistance $R_{0}$, in the magnitude of the gas-induced resistance change $∆R=R_{0}-R_{gas}$, and in the relative resistance response $Resp=\left(R_{0}-R_{gas}\right)/R_{0}$. Working towards MOX gas sensor systems with built-in self-test functionalities our focus in the following is on showing how these features can be detected and how their validity can be controlled under typical field conditions.

## 3. Gas Sensors Embedded in Micro-Reactor Systems

The key information provided by the above data is that the values of the relative resistance response are much better indicators of the momentary gas concentrations than the values of the sensor resistances themselves. The problem inherent in the evaluation of Resp values is that this quantity requires resistance measurements at two distinct points in time: a first measurement at time $t_{on}$ with the sensors operated under clean-air conditions, and a second one at time $t_{off}$ with the sensors still under analyte gas exposure. Whereas such measurements are easy to perform using gas test rigs, such measurements are principally impossible in field measurements where the sensors are continually exposed to the air ambient. In order to enable periodically repeated measurements under clean-air and analyte gas conditions also under field conditions, we report below on MOX gas sensors embedded into tiny ceramic micro-reaction chambers. When combined with a micro-pump such micro-reactor systems allow the MOX gas sensors to be periodically exposed to pure and analyte-containing air, thus enabling the evaluation of Resp values also under field conditions. 

**Figure 4:**
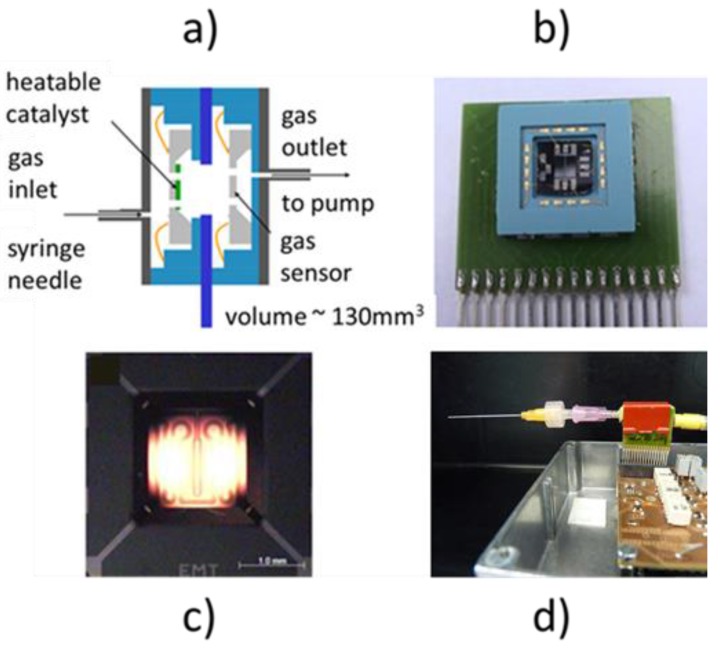
Enhanced micro-reactor system (EMRS); (**a**) cross section featuring embedded MOX gas sensor and heatable MEMS catalyst; (**b**) view onto MEMS micro-sensor; (**c**) view onto high-temperature heated catalyst; (**d**) EMRS mounted on printed circuit board featuring micro-pump and micro-reactor with hollow needle inlet and outlet hose connected to the pump.

Micro-reactor techniques have previously been applied to air monitoring problems [36,37]. In this early work traffic-related air contaminants (NO_2_, O_3_, CO, CH_4_) have been analyzed with the help of MOX gas sensors embedded in micro-reactor systems. In addition to their analytic capabilities, these systems also exhibited potential for introducing self-test capabilities. Although functionally successful, micro-reactor systems have found little general acceptance as the conventionally assembled [36] and MEMS-miniaturized [37] versions of such systems were relatively complex and incompatible with the low-cost approach generally followed in the MOX sensor field. Notable exceptions to this rule are references [38,39,40]. In order to reach wider acceptance of micro-reactor systems we introduce below a novel kind of micro-reactor which avoids the use of micro-valves, which had been the main contributors of complexity in our previous designs. This new kind of micro-reactor systems, which we call enhanced micro-reactor systems (EMRS) in the following, features four main components:

- A micro-chamber whose interior is separated from the ambient air by narrow gas inlet and outlet ports (Figure 4a,d);
- A MOX gas sensor embedded inside the micro-chamber (Figure 4b);
- A heatable catalyzer co-embedded inside the micro-chamber (Figure 4a,c);
- A micro-pump that allows the air inside the micro-chamber to be withdrawn and to be replaced by outside air that may or may not contain reactive gas components.

For the sake of gas analysis such EMRS are operated in a periodic manner, alternating between two different modes:

- In an actively forced flow-through mode (pump on);
- In an effectively separated no-flow mode (pump off).

During the actively forced flow phase, outside air with an unknown content of reactive trace gases is pumped into the micro-reactor at time $t_{on}$ and the flow is maintained up to time $t_{off}=t_{on}+∆t_{on}$. During this period of forced flow the MOX gas sensor is exposed to the sampled outside air with unknown content of analyte gas. At the end of this interval, the sensor resistance $R_{gas}\left(t_{on}+∆t_{on}\right)$ is measured. In the subsequent no-flow phase with duration $∆t_{off}$ the internal air volume is effectively separated from the external atmosphere due to the large flow resistances of the inlet and outlet ports. During this no-flow phase the sampled reactive gas components continue to interact with the heated MOX sensor surface thereby converting the reactive gas components into less reactive follow-on products, i.e., mainly H_2_O and CO_2_, which produce a reference gas atmosphere inside the micro-reactor [36,37]. The sensor resistance $R_{0}\left(t_{on}+∆t_{on}+∆t_{off}\right)$ developing in this reference atmosphere forms the reference point, against which the gas-induced resistance $R_{gas}$, developing during the next forced flow period, can be compared. At this point it is relevant to note that the value of $R_{0}\left(t_{on}+∆t_{on}+∆t_{off}\right)$ will still depend on the ambient humidity concentration as H_2_O, as an incombustible air component, does not take part in the reactor-internal conversion processes [36,37]. In case the drawn-in gas samples cannot be converted rapidly enough into reference air during the time $∆t_{off}$, the process can be accelerated by additionally heating the co-integrated catalyst.

The EMRS-based gas detection experiments reported below had been performed using Au (gold)-doped SnO_2_ thin films produced using a modified RGTO (rheotaxial growth and thermal oxidation) technique [41] and deposited on pre-fabricated MEMS heater elements [42,43] (Figure 4b). During the EMRS experiments, the embedded MOX sensors were heated to their operation temperature via their platinum (Pt) heater meanders and the Pt heater resistance values developing during the heating were used to infer the actual values of the sensor operation temperature. The co-integrated heatable catalyst was produced by the same MEMS technology as the MOX gas sensors. The catalyst elements, however, had a much larger heatable hotplate area, amounting to 1.5 mm × 1.5 mm [43] (Figure 4c). In order to make these elements catalytically active, thin films of palladium (Pd) were evaporated onto the back-surface of the hotplates. With the external micro-pump (Figure 4d) gas flow rates through the EMRS in the pump-on states of approximately 150 sccm could be realized. More details about the MEMS sensor and catalyst components can be found in Appendix B. 

## 4. Micro-Reactor-Based Gas Detection

**Figure 5:**
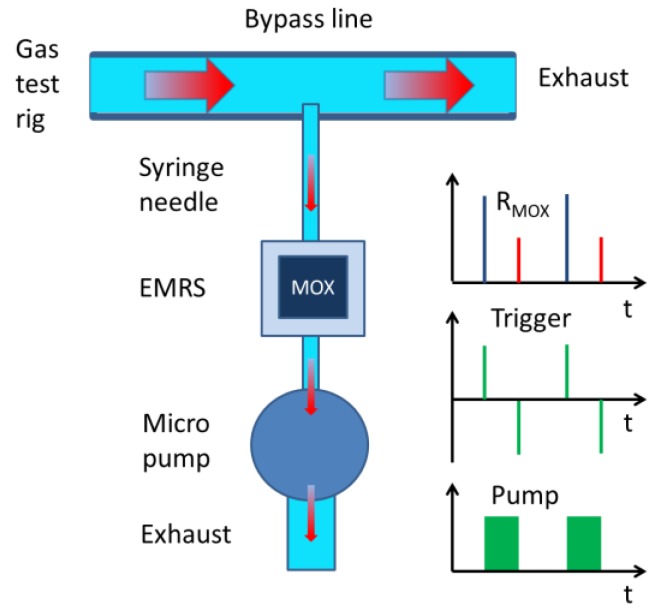
EMRS gas detection architecture. Synthetic air with analyte gases are dosed into the bypass line. The EMRS is fed with gas samples through pump-enforced gas streams. MOX resistances are read out during pump-on and pump-off trigger pulses and stored for later evaluation.

The functioning of an EMRS as a gas sensor system is illustrated in Figure 5. This figure in particular shows how the EMRS operation was investigated and demonstrated using a standard gas test rig. With this gas test rig dry synthetic air (SA)/analyte gas mixtures were prepared and passed down a bypass line and finally burnt down in the exhaust. Samples of the SA/analyte gas mixtures were extracted through a thin syringe needle into the EMRS system whenever the micro pump of the EMRS system was activated. During pump-on periods the micro-pump was activated to generate a gas flow of about 150 sccm/min, which allowed a high number of gas exchanges during typical sampling periods of 1 min or more. The main intention for using high flow rates was rapidly removing any traces of gas that were left inside the reactor at the end of each preceding no-flow period. The resulting MOX sensor resistance changes were read out with a rate of 1 Hz and the gas response patterns during pump-on periods were observed to be closely similar to those observed with non-embedded sensors. Those resistance values coinciding with the pump-on and pump-off trigger pulses at times $t_{on}$ and $t_{off}$ were stored for the evaluation of the relative resistance response values

$$Resp\left(t\right)=\frac{R_{0}\left(t_{on}\right)-R_{gas}\left(t_{off}\right)}{R\left(t_{on}\right)};\;t_{on}\leq t\leq t_{off}.$$

As we were interested in reducing gas detection, the sensor operation temperature was set at a relatively high value (~450 °C). At this temperature the used MOX gas sensors had a good sensitivity to H_2_ and hydrocarbons, but only a tiny response to NO_2_. 

As in our EMRS approach, the key piece of innovation is valve-less operation, we concentrate on the aspects of sampling, isolation and conversion of gas samples within the EMRS interiors, considering a limited range of gases (H_2_ and propyne), which are reasonably representative of the much larger group of combustible analyte gases [44]. 

**Figure 6:**
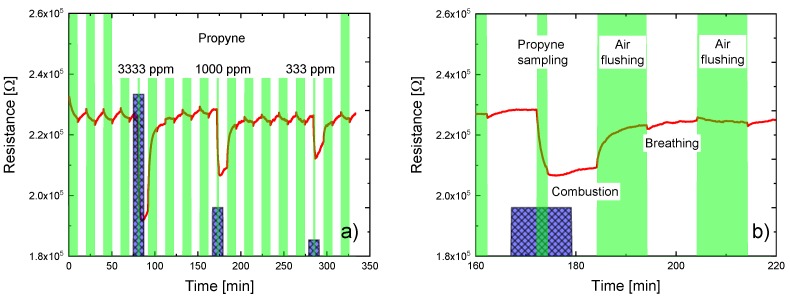
(**a**) (Green bars) timing of flow and no-flow periods of the EMRS; (blue bars) timing of propyne admixtures in the bypass line. Red line: reaction of MOX sensor resistance to pump-on and pump-off cycles and to gas sampling events; (**b**) Detail view onto the MOX sensor variation before, during and after a typical gas sampling event.

As a first result we demonstrate in Figure 6 that the special design of our EMRS allows environmental gas samples to be drawn into the EMRS and to be isolated there without the necessity of additional valves. In order to demonstrate this, the EMRS was periodically cycled between flow and no-flow periods by activating and de-activating the micro-pump. In Figure 6a the active flow periods are indicated by green vertical bars. Flow and no-flow periods typically lasted 10 min. During the first four flow/no-flow periods dry synthetic air was flown through the bypass line and partially sampled into the EMRS with the help of the micro-pump. Reference to Figure 6a shows that, during this first part of the experiment, the MOX sensor resistance undergoes small periodic changes. Similar periodic changes were also observed in the resistance of the Pt heater meander. With the known temperature coefficient of the Pt heater meander it could be inferred that the MOX gas sensor is experiencing temperature changes in the order of several degrees centigrade as it is cycled through subsequent flow and no-flow phases. Clearly, as MOX sensing layers also feature sizeable temperature coefficients of resistivity, thermally induced variations in the MOX sensor resistance are observed, which must not be misinterpreted as any form of gas response. A genuine gas response clearly occurs during the fifth time slot. During this time slot 3333 ppm of propyne gas was passed through the bypass line for a total period of 10 min. In the middle of this period the micro-pump was activated for 2 min to draw the propyne into the EMRS. Reference to Figure 6a shows a strong reducing gas response upon pump actuation. Thereafter, another four periods of flow/no-flow operation were carried out, this time sampling dry synthetic air again. During this phase the MOX sensor resistance recovers to baseline again, showing in the end the small thermally induced periodic resistance changes discussed before. In the 10th and 16th time slot additional propyne sampling processes were carried out, but at significantly lower concentrations. As expected, smaller reducing gas responses are observed at those occasions.

Figure 6b shows in more detail the MOX sensor resistance variations before, during and after a propyne gas sampling event. The first interesting observation is that—before pump actuation—the MOX sensor resistance remains at its baseline value when the bypass gas flow is changed from dry synthetic air to synthetic air containing 1000 ppm of propyne. This non-reaction clearly demonstrates the high degree of dynamic separation of the EMRS exterior and interior as long as the micro-pump is not activated. Upon pump-on, the MOX sensor resistance linearly decreases with time as more and more propyne is drawn into the EMRS interior and as it starts to interact with the heated MOX sensor surface. Upon pump-off, the MOX sensor resistance change is rapidly terminated, again demonstrating the efficient dynamic separation of the EMRS interiors and exteriors during no-flow periods. In the following no-flow period the sensor resistance slowly increases again as the trapped propyne is converted by surface combustion events into much less reactive H_2_O and CO_2_ molecules [36,37]. In the following flow period the remaining propyne is rapidly removed from the EMRS interior as the micro-pump again samples dry synthetic air from the bypass line.

**Figure 7:**
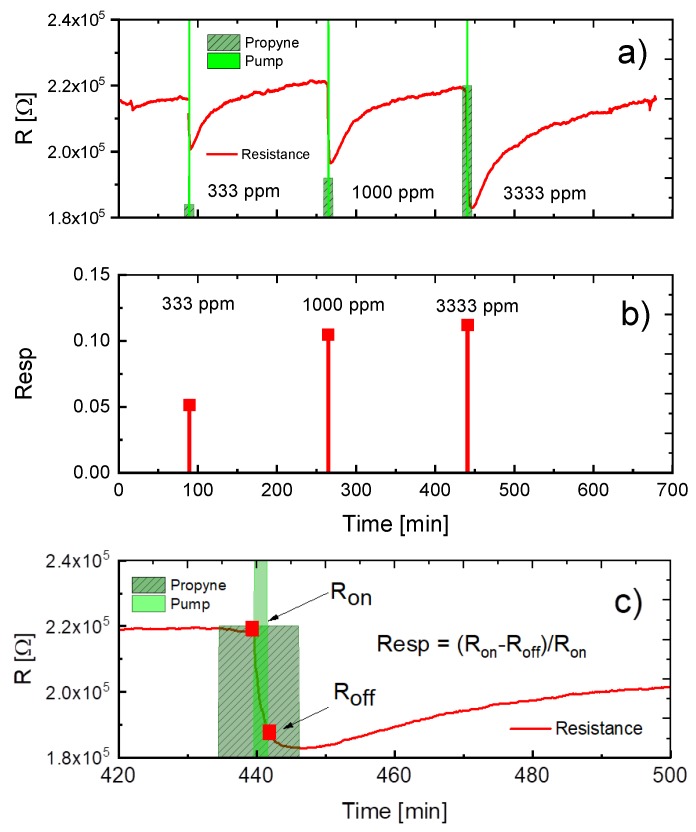
(**a**) Variation of MOX sensor resistance in response to three propyne sampling events; (**b**) relative resistance responses as evaluated from the sampling-induced MOX resistance changes in (**a**); (**c**) determination of the relative resistance response from the sensor resistance values observed upon pump-on and pump-off trigger events.

Figure 7 reports the results of another propyne sampling experiment. In this experiment three samples of propyne with increasing concentrations were sampled from the bypass line into the EMRS by operating the micro-pump for short two-minute intervals each. As expected and as shown in Figure 7a, in each of these sampling events the MOX sensor resistance drops by a concentration-dependent amount. The key difference with regard to the first experiment is that the successive sampling events were separated in time by more than two hours of no-flow operation to allow the MOX sensor to slowly return close to its baseline, simply by burning the propyne that had become trapped in the preceding sampling periods. The success of this latter experiment again emphasizes the efficiency of the dynamic separation of EMRS interiors and exteriors during no-flow phases and—as a second important result—the possibility of internal baseline restoration. Working from a reasonably well-defined baseline resistance the magnitudes of the gas-induced resistance changes in each sampling event can be clearly assessed and the relative resistance responses evaluated (Figure 7b). For clarity the process of the relative resistance determination is shown in greater detail in Figure 7c.

**Figure 8:**
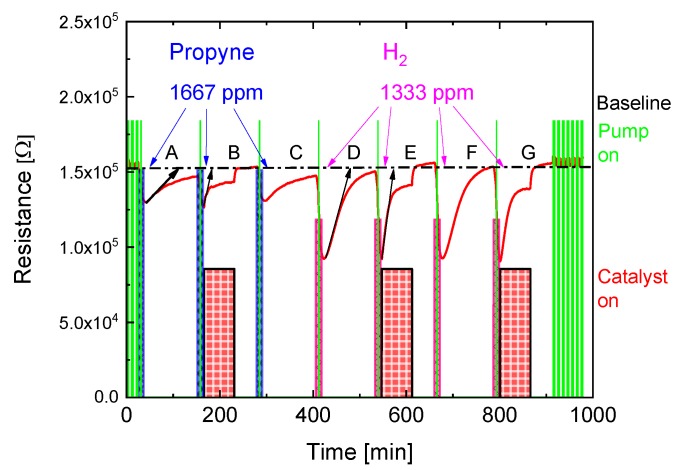
Variation of MOX sensor resistance in response to three propyne (A, B, C) and four H_2_ sampling processes (D, E, F, G). Catalyst activation (thick red bars) clearly enhances the speed of return to the sensor baseline resistance after reactive gas sampling. Return rates (black arrows) are seen to be gas-species dependent thus providing an additional measure of the reactivity of the sampled gas.

So far, we have been dealing with EMRS in which the built-in heatable catalyst has not yet been activated. In such EMRS the task of transforming reactive gas samples into incombustible reference gases is solely left to the MOX gas sensor with its limited size, temperature and catalytic activity. When the built-in catalyst is activated, the rate of return to baseline resistance can be considerably enhanced. This latter effect is demonstrated in Figure 8. In this experiment three propyne gas sampling processes (A–C) were carried out, followed by four processes in which H_2_ was sampled (D–G). In processes B, E and G the heatable catalyst was additionally heated to 500°C. In these latter processes the initial rise of the MOX sensor resistance in the post-sampling no-flow periods was considerably steeper than in the un-activated reference processes A, C, D and F. At first sight, a lower baseline resistance appears to be reached when the catalyst is on. A return to the original baseline level, however, does occur when the catalyst is switched off. When this is done, the excess heat inside the EMRS is dissipated and the sensor operation temperature is allowed to return to its original level. Additionally, it has to be taken into account that the catalyst operation will also raise the pressure inside the micro-reactor and thus cause inside air to be pushed out. As upon catalyst switch-off the internal pressure will decrease again, some outside air will be drawn in. As in the present experiment this switch-off had been performed at a time at which dry synthetic air was flowing in the bypass line, the return to baseline could also have been facilitated by the uptake of some additional fresh air. Possibly we are seeing at this point a limit to the valve-less dynamic separation of inside and outside air volumes. Future EMRS designs with more carefully designed flow resistances are likely to ameliorate this situation. 

## 5. Poisoning Detection

**Figure 9:**
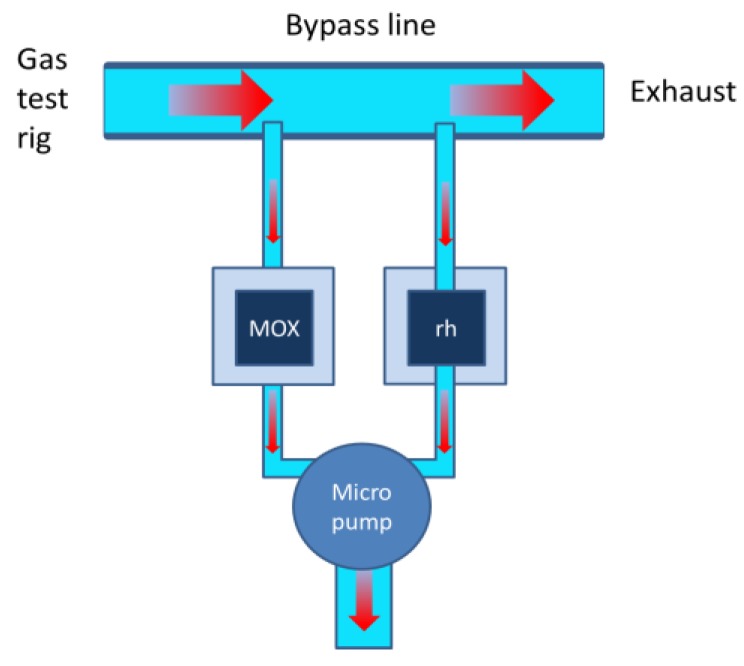
Self-testable EMRS sensor array featuring MOX gas sensor and a capacitive humidity (rh) reference sensor.

Whereas the self-test procedures discussed above allow the relative resistance responses to a particular gas to be detected, the determined values of Resp do not reveal those gas concentrations that have actually triggered the particular response. What also remains unclear is whether the magnitude of the responses might have changed over time due to some form of sensor degradation. In this context, measurements relative to known concentrations of reference gas are required. Additionally, measurements are required with a second sensor that did not suffer any degradation over time. As mentioned in chapter two, a reference gas that comes for free is water vapor. Water vapor is present in almost any conceivable application scenario in variable and in time-dependent concentrations. A micro-reactor arrangement which, in principle, allows such reference measurements to be performed is sketched in Figure 9. In Figure 9 we combine the MOX gas sensor to be self-tested with a humidity sensor that is resilient to the degradation processes that might deteriorate the performance of the MOX gas sensor. Both sensors are embedded in their own micro-reactor chambers to avoid thermal and chemical cross-talk between sensors. Alternation between flow- and no-flow conditions is performed by a common micro-pump. Upon pump-on, outside air is drawn through both chambers. Whereas the MOX gas sensor responds to the unknown outside atmosphere with a sensor resistance change, the humidity sensor simply measures the ambient relative humidity level. This latter measurement, of course, requires that the humidity sensor is completely selective to water vapor. Upon pump-off, both micro-reactors are sealed. Whereas in the humidity reference chamber the humidity will simply stay constant at its ambient air value, the trapped gases inside the MOX micro-reactor chamber interact with the heated MOX surface and get converted into less reactive follow-on products. Whereas H_2_, a wide range of hydrocarbons and CO, for instance, get converted into CO_2_ and H_2_O, the ambient humidity that had become co-trapped with the reactive gases will simply stay unconverted [36,37]. As CO_2_ is not detectable by MOX gas sensors, the saturation resistance of the MOX gas sensor will be close to the resistance level corresponding to the humidity level in the ambient air. In case the measured ambient humidity levels and the final MOX sensor resistance are stored and in case this same procedure is repeated over some time in different ambient humidity levels, the MOX sensor resistance can be assessed as a function of the humidity level in the ambient air. By comparing this measured function to a calibration line that had been established in the virgin state of this same sensor, possible degradation-induced changes in the gas response can be detected. In order to be successful, this procedure obviously requires the existence of humidity sensors that are completely selective to humidity and which are invulnerable to those reactive species that cause MOX sensor degradation.

**Figure 10:**
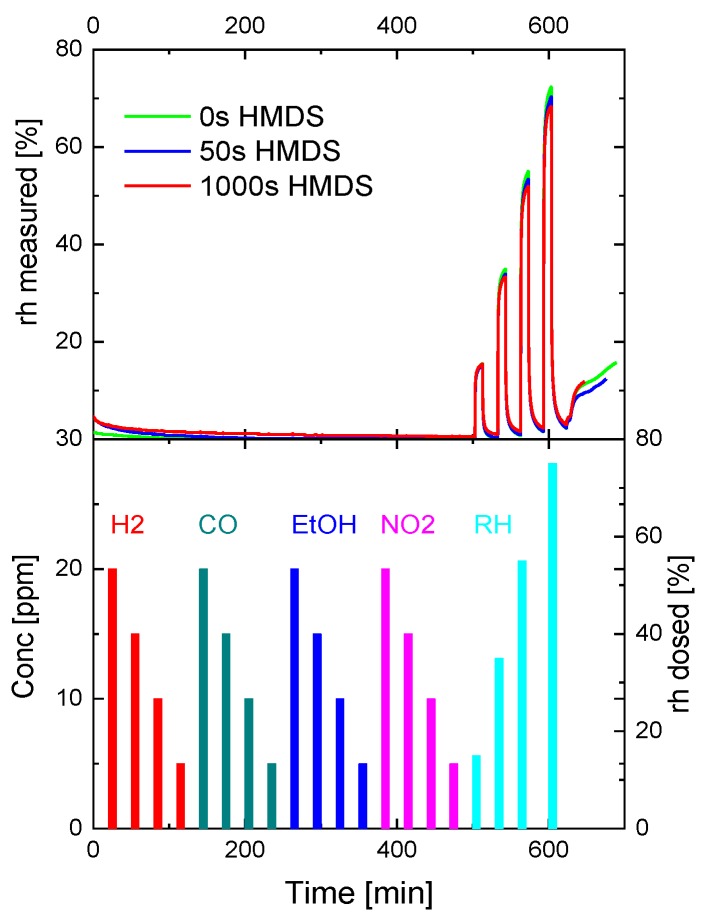
Response of a commercial capacitive humidity sensor after increasingly longer HMDS treatment times (**top**) to the gas concentration profiles shown at the (**bottom**).

That such invulnerable sensors actually might exist is suggested by the data in Figure 10. The data shown there firstly prove that room-temperature operated capacitive humidity sensors [45] are completely selective to water vapor. This excellent sensitivity derives from the fact that capacitive humidity sensors respond to the high dielectric constant (DK) of adsorbed water ($\varepsilon_{H2O}\approx 81$), which is far beyond the DK of almost all other reactive gases of interest. The second important observation is that capacitive humidity sensors are extremely resilient to prolonged HMDS poisoning. The data in the top panel of Figure 10 show that these feature practically the same humidity response both in their virgin states and after 1000 s exposure to high-concentration HMDS vapors. MOX gas sensors, in comparison, suffer from severely degraded responses after a few seconds of HMDS only. The immunity of capacitive humidity sensors to HMDS poisoning obviously derives from the fact that these are room-temperature operated and thus unable to provide the thermal energy input for the transformation of adsorbed HMDS vapors into dense passivation layers. As a consequence, heated MOX gas sensors suffer much more severely from HMDS vapors than the capacitive humidity sensors.

## 6. Possibilities and Limitations of the Micro-Reactor Approach

On the above pages we have demonstrated two key technological components that have considerable potential for arriving at self-testable MOX gas sensor systems. These are valve-less micro-reactor systems with embedded MOX gas sensors and highly selective and poisoning-resistant humidity sensors. As a test case for demonstrating the necessity of in-field Resp determinations and poisoning detection we have chosen the sensor degradation process of HMDS passivation of MOX sensor surfaces.

In service, gas sensor systems may encounter a virtually unlimited variety of substances that need to be detected or which may damage the sensor functionality. Any piece of research, however, needs to concentrate on a limited number of probable species and application cases. In this work we have concentrated on the detection of reducing gas species, in particular H_2_ and hydrocarbons which entirely convert into CO_2_ and H_2_O at heated MOX surfaces. Substances like NO_2_ or hydrocarbons with fluorine-, chlorine- or sulfur containing functional groups will, in addition to CO_2_ and H_2_O, also give rise to strong acid formers at the end of the conversion process. These acid-formers together with the ambient humidity are likely to pose problems which will require measures beyond those demonstrated here.

Considering the current state of the art and the range of substances considered in this work, two operational modes of microreactor-embedded gas sensors are conceivable:

(i) a normal observational mode, and
(ii) a self-test mode.

In the first mode, which essentially follows the procedures demonstrated in Figure 6, gas samples are repeatedly drawn from the environment into the EMRS and the values of $R_{gas}\left(t\right)$ at the end of each sampling period are recorded and compared to those sensor resistance values $R_{gas}\left(t-∆t\right)$ attained at the end of each preceding sampling period. The rate at which successive samples can be taken is relatively high and is mainly determined by the EMRS volume and the externally generated pump flow. In this mode of operation, essentially un-converted gas samples inside the EMRS are simply replaced by new ones and any eventual changes in the sensor resistance values $∆R_{gas}=R_{gas}\left(t\right)-R_{gas}\left(t-∆t\right)$ are used to calculate values of Resp which do not reference the sensor resistance at time t to its humidity-dependent baseline value at time t but to the gas-induced sensor resistance $R_{gas}\left(t-∆t\right)$ attained at the end of each preceding sampling period:

$$Resp\left(t\right)=(R_{gas}\left(t-∆t\right)-R_{gas}\left(t\right))/R_{gas}\left(t-∆t\right).$$

As demonstrated in the discussion of §2 and also in one of our recent papers [46], this method of Resp processing of sensor resistance data tends to remove the effects of gas concentration pile-ups either on the sensor surface or in the periphery of the sensors from the directly observed resistance data. Due to this compensation effect, EMRS operation in the normal observational mode clearly generates added value relative to conventionally operated non-embedded gas sensors. 

In case indications do arise that poisoning interactions might have occurred over time or in case pre-defined service intervals are reached, a transition to the self-test mode of EMRS operation is indicated. In this second mode of operation, which essentially follows the procedures demonstrated in Figure 7, significantly more time needs to be spent in between successive sampling events. During this time entrapped gas samples are completely converted into CO_2_ and H_2_O and the sensor resistance is able to relax back to its baseline value corresponding to the momentary environmental humidity level. As argued in Section 5, these saturation levels of sensor resistance can be compared to the calibration values established in the virgin state of this same sensor. Tests of this kind, of course, need to be performed at times in which a continuous monitoring activity is not required. Any failures of observing conversion curves in this self-test mode provide indications into the direction of sensor failure, sensor degradation or catalyst inactivation. In such events the sensor user should be alerted that sensor data can no longer be trusted and that some kind of service is indicated or that a sensor replacement should be carried out.

Concerning response times, the currently demonstrated conversion times of the order of one hour are by no means a limit. A significant potential for cutting down conversion times is reducing sensor and reactor sizes. In the current technology a very obvious measure of reducing reactor volumes is decreasing the dead volume between micro-reactor and micro-pump. In principle such a reduction can be achieved by connecting the micro-reactor and the micro-pump through another syringe-type flow limiter. In future MEMS-miniaturization steps the syringe-type entrance and exit channels could also easily be integrated into a silicon lid that seals the micro-reactor interior from the environment. Extremely fast conversion times could in principle be achieved when single-nanowire gas sensors [16,17] are embedded into reactors of similar nanometric size.

Looking beyond the limits of sensor self-test and completely staying within the limits of the current state of the art, an important and completely unexploited potential of micro-reactor embedded gas sensors is in the area of gas analysis where precision and selectivity is required rather than high-rate monitoring. In this range of applications the key pieces of information are those conversion curves that are observed in the no-flow phase. Work on pellistor gas sensors [42], for instance, has shown that such conversion functions are highly gas-species-dependent. From this experience it can be extrapolated that the observed conversion functions provide information about the reactivity of the entrapped gas samples and thus useful analytical information. 

## Appendix A

SnO_2_ nanowires were been synthesized by means of evaporation-condensation method according to the protocol described elsewhere [33,34]. Briefly, alumina substrates (2 mm × 2 mm × 0.25 mm in size) are pre-patterned with Pt acting as catalyst for the growth of nanowires. SnO_2_ powders are placed in a tubular furnace and heated at a temperature of 1370 °C in a background of 100 mbar of Ar; substrates are lodged downstream in a colder region at around *T* = 800 °C. Thereafter, Pd nanoparticles are sputtered at room temperature over the nanowire mesh by means of RF magnetron sputtering up to a Pd/Sn ratio of 3(wt %.). Pt electrodes with a comb-like shape and a gap of 200 nm are deposited over the tin oxide layer to provide contacts for two-probe electrical measurements. A Pt meander, acting both as a heater resistance and a temperature sensor is sputtered on the rear side of the substrate. Finally, the device is soldered on a commercial TO38 case. Full details of the sensor layout are provided in [35].

## Appendix B

The MEMS microheaters were fabricated by a combination of dry and wet etching techniques starting from silicon-on-insulator (SOI) wafers [44,45]. The microstructuring leads to free-standing silicon bridges suspended over a KOH etch groove. Pt electrodes sputtered onto the front side of the silicon bridges served as heater meanders and temperature sensors and as bottom contacts for SnO_2_ thin films. Thin films of SnO_2_ were deposited onto pre-processed MEMS microheaters by means of the RGTO (rheotaxial growth and thermal oxidation) technique [43]. The preparation of catalytically enhanced SnO_2_ starts with the evaporation of a three-layer stack of Sn/Au/Sn onto a pre-fabricated MEMS heater substrate. After evaporation the metallic films are annealed in ambient air at a temperature of 600 °C for several hours to completely convert the tin (Sn) into nano-crystalline SnO_2_ films [43]. During oxidation nano-crystalline SnO_2_ films with an average grain size of about 20 nm are formed, alongside with interspersed Au catalyst clusters, which phase-separate from the emerging SnO_2_ during the tin oxidation process.
